# Proteomic Analysis of Methanococcus voltae Grown in the Presence of Mineral and Nonmineral Sources of Iron and Sulfur

**DOI:** 10.1128/spectrum.01893-22

**Published:** 2022-07-25

**Authors:** Katherine F. Steward, Devon Payne, Will Kincannon, Christina Johnson, Malachi Lensing, Hunter Fausset, Brigitta Németh, Eric M. Shepard, William E. Broderick, Joan B. Broderick, Jen Dubois, Brian Bothner

**Affiliations:** a Department of Chemistry and Biochemistry, Montana State University, Bozeman, Montana, USA; b Department of Microbiology and Cell Biology, Montana State University, Bozeman, Montana, USA; University of Massachusetts Amherst

**Keywords:** methanogen, iron-sulfur cluster, proteomics, pyrite, mackinawite

## Abstract

Iron sulfur (Fe-S) proteins are essential and ubiquitous across all domains of life, yet the mechanisms underpinning assimilation of iron (Fe) and sulfur (S) and biogenesis of Fe-S clusters are poorly understood. This is particularly true for anaerobic methanogenic archaea, which are known to employ more Fe-S proteins than other prokaryotes. Here, we utilized a deep proteomics analysis of Methanococcus voltae A3 cultured in the presence of either synthetic pyrite (FeS_2_) or aqueous forms of ferrous iron and sulfide to elucidate physiological responses to growth on mineral or nonmineral sources of Fe and S. The liquid chromatography-mass spectrometry (LCMS) shotgun proteomics analysis included 77% of the predicted proteome. Through a comparative analysis of intra- and extracellular proteomes, candidate proteins associated with FeS_2_ reductive dissolution, Fe and S acquisition, and the subsequent transport, trafficking, and storage of Fe and S were identified. The proteomic response shows a large and balanced change, suggesting that M. voltae makes physiological adjustments involving a range of biochemical processes based on the available nutrient source. Among the proteins differentially regulated were members of core methanogenesis, oxidoreductases, membrane proteins putatively involved in transport, Fe-S binding ferredoxin and radical S-adenosylmethionine proteins, ribosomal proteins, and intracellular proteins involved in Fe-S cluster assembly and storage. This work improves our understanding of ancient biogeochemical processes and can support efforts in biomining of minerals.

**IMPORTANCE** Clusters of iron and sulfur are key components of the active sites of enzymes that facilitate microbial conversion of light or electrical energy into chemical bonds. The proteins responsible for transporting iron and sulfur into cells and assembling these elements into metal clusters are not well understood. Using a microorganism that has an unusually high demand for iron and sulfur, we conducted a global investigation of cellular proteins and how they change based on the mineral forms of iron and sulfur. Understanding this process will answer questions about life on early earth and has application in biomining and sustainable sources of energy.

## INTRODUCTION

All cells require iron (Fe) and sulfur (S) as essential components of amino acids, vitamins, coenzymes, and cofactors (1). Specifically, S is utilized in cysteine and methionine, and both Fe and S are required for heme and biological Fe-S clusters, which function in electron transfer, substrate binding, and a wide range of enzyme catalysis (2). The ability to obtain Fe and S from the environment is critical for the growth of microorganisms. Under aerobic conditions, the predominant form of Fe is Fe(III) in low-solubility iron oxides; in order to acquire this iron, microorganisms synthesize chelating ligands known as siderophores that bind and solubilize Fe(III), making it available to specific transport proteins (3). Sulfur can be acquired as sulfates, sulfites, or thiosulfates, or as organic molecules such as cysteine, and can then be converted to other forms enzymatically (4). Under anaerobic conditions, the mechanisms of Fe and S acquisition are less well understood, although Fe(II) and reduced forms of sulfur would be the predominant forms of these elements. Given the abundance of iron-sulfur minerals such as pyrite (FeS_2_) on the early Earth prior to the advent of oxygenic photosynthesis, as well as in extant anaerobic environments, it is interesting to consider whether anaerobic microorganisms such as methanogens might acquire Fe and S directly from mineral sources (5–7).

Methanogens are a deeply rooted branch of archaea that produce methane as a by-product of their central metabolism (8). This metabolic process is catalyzed by enzymes that require Fe-S cofactors (9, 10). Methanogens also have Fe-S-containing metalloenzymes that are capable of using key metalloclusters to catalyze oxidation-reduction reactions and to carry out electron transfer, Fe and S storage, small-molecule activation, and a range of other reactions (11–14). Examples of important enzyme systems that rely on Fe-S clusters yet evolved before biological oxidation of the environment include hydrogenases, nitrogenases, and methane-generating enzymes (15). In addition to these systems, much of the supporting biochemistry requires cells to have steady access to soluble Fe and S. In fact, methanogens may rely on iron more than typical aerobic microbes: studies comparing the Fe content of Escherichia coli and Methanococcus maripaludis revealed that *M. maripaludis* uses 15-fold more Fe than E. coli per milligram of protein (16).

Methanogens are among the most primitive of extant organisms (6, 8, 17). They can be divided into two lineages based on the presence or absence of a SufS gene, which codes for a cysteine desulfurase that is required to liberate S from cysteine (18). The ancestral lineage (class I) does not have SufS. Recently, methanogens were shown to reduce pyrite (FeS_2_) and use Fe-S by-products to meet biosynthetic demands (19). Further, it was found that Methanococcus voltae cells contain 167% more Fe when grown on FeS_2_ than ferrous Fe [Fe(II)] and sulfide (HS^−^) (20). Therefore, Fe and S in FeS_2_ are bioavailable to at least some microorganisms in anoxic environments, forcing a reevaluation of modern and ancient biogeochemical cycles. The mechanism and cellular pathways responsible for this process have yet to be elucidated. Recent transcriptomics work on Methanosarcina barkeri cultured in the presence of different Fe/S sources implicates alpha-keto reductases, a flavin mononucleotide-dependent flavodoxin reductase, and hydrolases as putative enzymes involved in FeS_2_ reduction (21). Our previous work with M. voltae showed that cells grown on FeS_2_ are smaller and may use an IssA protein to sequester iron as a thioferrate-like species (20). We also concluded that methanogenesis pathways operate similarly when the organism is grown on FeS_2_ or Fe(II) and HS^−^.

Here, we extended the prior study by using M. voltae A3 to gain insight into the global changes to proteins and pathways critical for reductive dissolution of FeS_2_ and assimilation of Fe and S by-products by analyzing samples prepared from both FeS_2_ and canonical Fe(II)/HS^−^ culture conditions. The differential analysis highlighted proteins potentially mediating uptake of Fe and S (presumably as soluble aqueous FeS [FeS_aq_] molecular clusters), membrane proteins involved in transport, as well as the intracellular partners that are involved in the storage of Fe and S and the subsequent assembly of Fe-S clusters. These experiments also shed light on cell-wide changes in protein synthesis, energetic strategies, and metabolic priorities. Our in-depth liquid chromatography-mass spectrometry (LCMS)-based proteomics analysis of the intra- and extracellular proteomes under different conditions captured 77% of the predicted protein-coding regions of M. voltae. Widespread changes in the intra- and extracellular protein pools demonstrate that this methanogen is sensitive to the available form of Fe and S, as indicated by changes in a wide range of metabolic, oxidoreductase, ribosomal, and transport proteins in response to available Fe and S species.

## RESULTS

### Global intracellular proteomics.

To elucidate proteins involved with assimilation of Fe and S and to determine if pathways varied when different sources of Fe and S were supplied (mineral versus nonmineral), M. voltae A3 cells were grown in minimal base salts medium provided with either 20 μM Fe(II) and 2 mM HS^−^ or sufficient synthetic FeS_2_ to provide the equivalent of 2 mM S, as the sole source of Fe and S. The intracellular and extracellular protein fractions were analyzed separately by shotgun proteomics to identify and quantify changes in cellular protein expression.

**FIG 1:**
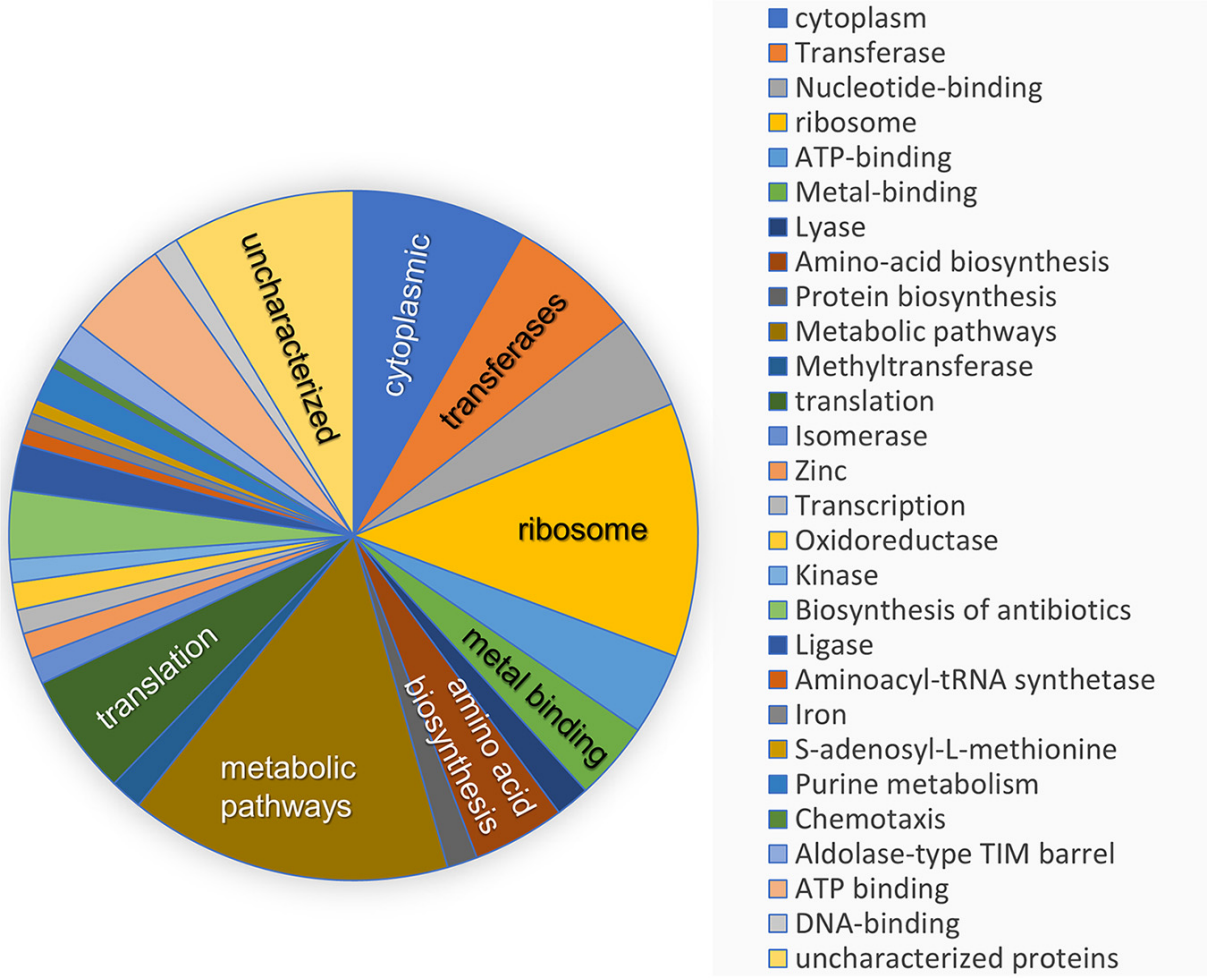
Pathway distribution of identified intracellular proteins based on gene annotations in DAVID (23, 24).

**FIG 2:**
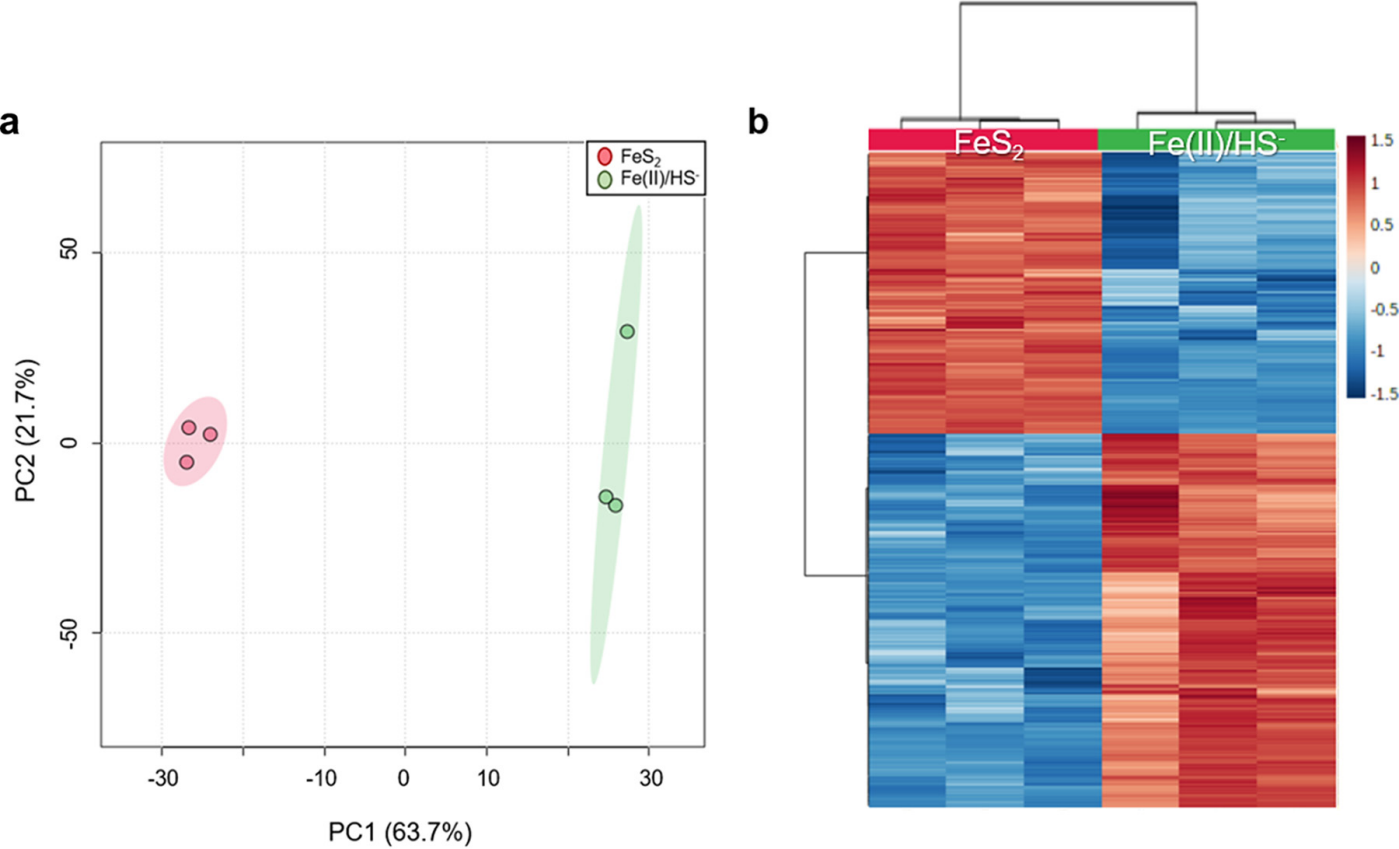
Differential analysis of sample groups. (a) Principal-component analysis (PCA) of FeS_2_ and Fe(II)/HS^−^. (b) Heat map based on the top 500 proteins that differentiate cells grown on FeS_2_ from those grown on Fe(II)/HS^−^ as the mineral source. Biological replicates (columns) and proteins (rows) are arranged by hierarchical clustering using Euclidean distance and Ward clustering algorithms. The key indicates fold change in protein abundance.

All samples shared 1,269 of 1,658 (77%) protein-coding genes (22) predicted from the M. voltae A3 genome. Grouping the identified proteins by pathway (as identified by DAVID analysis [23, 24]) showed broad coverage of functional classes, as expected given the high percent coverage of the genome (Fig. 1). A principal-component analysis (PCA) was applied to make an unsupervised analysis of the sample sets. The two experimental groups were well separated in the first dimension and with samples clustering much more closely within groups than between groups (Fig. 2). Comparative analysis of normalized protein abundances under the two growth conditions [Fe(II)/HS^−^ versus FeS_2_] using Student’s *t* test showed that 509 proteins had significantly different abundances (fold change [FC] > 2; *P < *0.05). Two hundred eighty-five of these proteins more abundant in the Fe(II)/HS^−^ condition, and 224 proteins were more abundant in cells grown on FeS_2_ (see Tables S1 and S2 in the supplemental material). Hierarchical clustering was used to compare the proteomic response of M. voltae cells grown under the two conditions. When the 500 most differentially expressed proteins were considered, samples clustered by treatment displaying a consistent response to the Fe and S source (Fig. 2b). To gain insight into the roles of the differentially expressed proteins, we assigned functional annotations to each using Gene Ontology (GO) categorization (Table 1). M. voltae cells grown with Fe(II)/HS^−^ tended to have a greater abundance of proteins associated with amino acid, protein, and nucleic acid metabolism, ribosomal proteins, membrane transport, and cofactor biosynthesis than cells grown with FeS_2_. Proteins with potential roles in Fe and S metabolism were detected in higher abundance in the FeS_2_ samples, including metal uptake, trafficking, and storage proteins as well as transcriptional regulators and oxidoreductases. It is important to note that 22% (111) of the regulated proteins lacked an assigned GO category, with 62% (69 proteins) of these in the FeS_2_ group, emphasizing that some proteins involved in growth on FeS_2_ are uncharacterized.

**TABLE 1:** GO annotation

Function and protein	No. more abundant in:		No. with no change
	FeS_2_	Fe(II)/HS^−^	
Cellular maintenance			
Metal uptake, trafficking, storage	10	5	
Transcriptional regulation and/or signaling	12	9	7
Uncharacterized Fe-S binding proteins	6	3	
ABC transporters^*a*^	4	5	6
Ferredoxins	14	7	18
Sulfur metabolism^*a*^	0	2	6
Radical SAM proteins	5	9	13
Oxidoreductases	27	12	
Nitrogenase (not present in M. voltae)^*a*^	**3**	**4**	
Hydrogenase (includes F420 dependent)	8	5	2
Metabolic pathways	1	1	10
Glycolysis/gluconeogenesis	0	1	7
TCA cycle proteins^*a*^	1	2	11
Methanogenesis/methanogenesis marker^*a*^	6	5	35
Uncharacterized proteins	37	35	126
Biosynthetic pathways	13	13	
Cofactor biosynthesis	12	20	3
Amino acid/protein metabolism	13	28	1
Carbohydrate metabolism	8	5	
Transferases, lyases, ligases	3	10	9
Hydrolases: esterases, amidases	8	3	3
Nucleotide binding, kinase, phosphatases	4	10	3
Nucleic acid metabolism	12	51	19
Conjugation, cell division	0	1	1
Motility	0	6	1
Chemotaxis	0	2	6
Ribosomal protein	1	39	40
S-layer, cell wall protein	3	3	5
Transport	3	5	
Membrane proteins	5	2	16
Membrane transport	3	9	3
CRISPR	4	1	12
Stress-related proteins	19	16	31
Respiration	0	3	6

a Pathways of interest.

**FIG 3:**
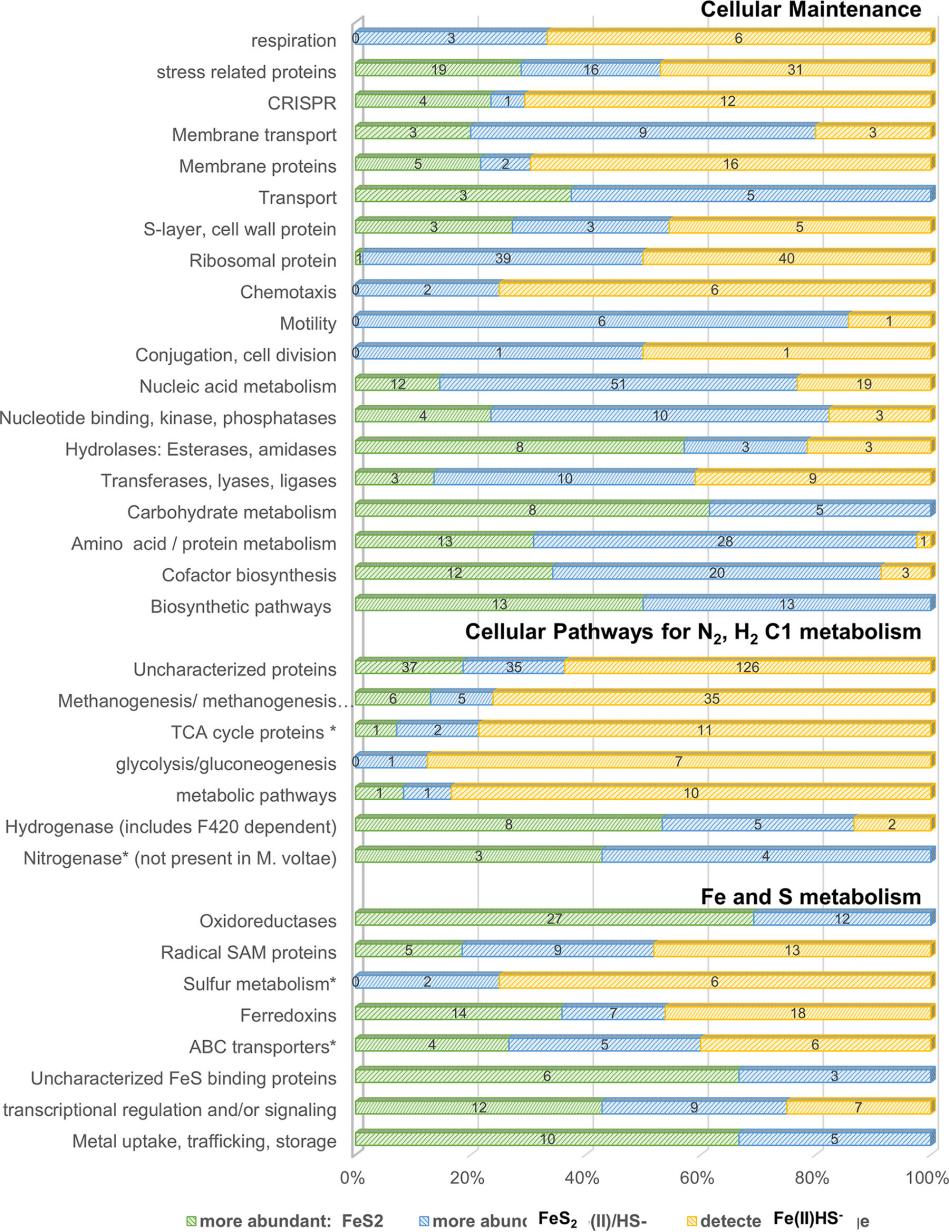
Overview of pathway specific changes with respect to culture conditions. Colored segments show the percentage of intracellular proteins in a given pathway that were more abundant in the presence of FeS^2^ (green), more abundant in the presence of Fe(II)HS^−^ (blue), and unchanged (yellow). Numerals on the bars show the actual number of proteins. Annotations were made using UniProt, GO, STRING, and KEGG.

Next, we investigated specific metabolic pathways to develop a deeper understanding of the physiological demands imposed by the different Fe and S sources. We first looked at core metabolic pathways, including the tricarboxylic acid (TCA) cycle, glycolysis, and methanogenesis as annotated in KEGG (25). For the most part, there was little change (Fig. 3 and Table 2). The higher abundance of proteins associated with amino acid metabolism, nucleic acid metabolism, and cofactor biosynthesis in the Fe(II)/HS^−^ cultures suggests a phenotype which dedicates resources to maintenance and growth, compared to FeS_2_ cultures. Consistent with this idea is the greater representation of proteins with ATP binding domains in Fe(II)/HS^−^-grown cells compared to FeS_2_-grown cells (Fig. 3). Proteins predicted to have a role in nitrogenase-like pathways and nitrogen cycling had similar abundances except for two proteins with similarity to NifB and more specifically the IssA clade (Mvol_0693 and Mvol_0689) that were more abundant in the FeS_2_ condition and a NifH homolog that was also significantly higher in the FeS_2_ condition. NifH is required for the synthesis of cofactor F430, which is needed by methyl coenzyme M reductase (26). While M. voltae fails to grow diazotrophically, there are proteins annotated as nitrogenase-like. Investigation of these homologs found them to be similar to an IssA protein related to Fe storage as intracellular thioferrate nanoparticles (20, 27). Proteins associated with methanogenesis were compared to assess changes to the central energy metabolism of M. voltae (6, 28). Eleven of the 46 proteins (*P* < 0.05) associated with methanogenesis were significantly different between conditions, showing a balanced response (Fig. 3; Tables 1 and 2). While there was not a robust response in either direction, the regulation of methanogenesis marker proteins, as annotated by UniProt (29), shows that there could be differences in core energy production for this methanogen between the two growth conditions.

**TABLE 2:** KEGG annotation

Category and protein	Accession no.	Intracellular abundance (log) in:		Fold change^*a*^	*P* value
		FeS_2_	Fe(II)/HS^−^		
Sulfur metabolism					
SufBD	D7DT52	1.193220	1.089336		
Phosphoadenosine phosphosulfate reductase	D7DRD1	0.067645	0.209334	−3.09454	0.0017082
SufC	D7DT53	0.567117	0.401460		
TCA cycle					
Thiamine pyrophosphate protein domain protein TPP-binding	D7DUC1	1.46105	1.59608		
Pyruvate flavodoxin/ferredoxin oxidoreductase domain	D7DUC2	3.43884	5.41021		
Acetyl-CoA carboxylase, biotin carboxylase	D7DR99	0.47494	1.20170	−2.53024	5.63E−05
Pyruvate flavodoxin/ferredoxin oxidoreductase domain	D7DVA0	0.77251	0.83106		
Oxaloacetate decarboxylase alpha subunit	D7DRA0	1.27881	4.07144	−3.18380	1.58E−06
Thiamine pyrophosphate protein domain protein TPP-binding	D7DRF8	0.25659	0.27201		
Malate dehydrogenase [NADP(+)]	D7DTV0	0.19713	0.38894		
Pyruvate ferredoxin/flavodoxin oxidoreductase, delta subunit	D7DUC3	0.47768	0.57191		
Pyruvate/ketoisovalerate oxidoreductase, gamma subunit	D7DUC4	2.18007	2.06684		
FAD-dependent pyridine nucleotide-disulfide oxidoreductase	D7DT22	0.05533	0.00596	9.2755	0.000356
Pyruvate/ketoisovalerate oxidoreductase	D7DRF7	0.80107	0.76887		
Hydro-lyase, Fe-S type, tartrate/fumarate subfamily, beta subunit	D7DUG7	0.03225	0.03141		
Hydro-lyase, Fe-S type, tartrate/fumarate subfamily, alpha subunit	D7DV42	0.06394	0.09762		
Sulfur relay system					
DsrE family protein	D7DUT0	0.146182	0.135039		
SirA family protein	D7DUT1	0.025526	0.050366		
Molybdopterin biosynthesis MoaE protein	D7DTH9	0.127704	0.064877		
UBA/THIF-type NAD/FAD binding protein	D7DSD7	0.048156	0.028839		
Thiamine S protein	D7DT40	0.009235	0.012298		
Molybdopterin converting factor, subunit 1	D7DU80	0.102335	0.072400		
ABC transporter-related proteins					
Extracellular solute-binding protein family 1	D7DV05	2.89244	2.05020		
5-Formaminoimidazole-4-carboxamide-1-(beta)-d-ribofuranosyl 5′-monophosphate synthetase	D7DS15	11.55548	5.51428		
ABC transporter-related protein	D7DTS1	0.06359	0.01663	3.8233	0.0023623
ABC transporter-related protein	D7DR50	0.23008	0.55915	−2.43019	0.0003957
ABC transporter-related protein	D7DT53	21.68802	15.94546		
Formate/nitrite transporter	D7DTM1	1.57738	1.21316		
Substrate-binding region of ABC-type glycine betaine transport system	D7DRC3	0.52625	0.38597		
ABC transporter-related protein	D7DUU7	0.10431	0.27874	−2.67215	0.0057287
Glycine betaine/l-proline ABC transporter, ATPase subunit	D7DRC5	0.11761	0.16812		
ABC transporter-related protein	D7DTC2	0.00454	0.02613	−5.76203	0.0038215
Molybdenum ABC transporter, periplasmic molybdate-binding protein	D7DTE8	0.00439	0.04525	−10.30237	0.013507
Phosphate-binding protein	D7DSZ3	0.03425	0.03782		
Methanogenesis proteins					
MfrA	D7DTS3	6.79839	5.29676		
MfrB	D7DUW6	4.56420	5.12853		
MfrC	D7DTS2	3.71482	3.51635		
MfrE	D7DTC5	0.11912	0.42939	−3.6046	0.021716
MfrA2	D7DTE0	0.09261	0.07055		
MfrC2	D7DTE1	0.11450	0.05754		
MfrB2	D7DTE2	0.06535	0.03133	2.0857	0.016285
Ftr	D7DQM5	6.53255	7.18286		
Mch	D7DSC8	4.17309	3.99639		
Mtd	D7DR53	21.68802	15.94546		
Mer	D7DV70	15.61439	7.61597	2.0502	0.0056952
MtrA	D7DUI1	0.08459	0.25803		
MtrD	D7DUH7	0.00019	0.01764		
MtrG	D7DU12	1.34331	0.24835		
MtrB	D7DUH9	1.08177	1.52612		
MtrC	D7DUH8	0.09097	0.17195		
MtrH	D7DUI3	0.74917	0.25003		
McrG	D7DUH4	6.19291	7.00046		
McrA	D7DUH5	11.52656	8.86821		
McrB	D7DUH1	13.73103	7.97736		
FrhG	D7DSY8	0.08514	0.03158		
FrhA	D7DU38	20.24111	10.02758	2.0185	0.0009404
FrhB	D7DU35	6.66821	3.28590	2.0293	0.0013333
HdrA	D7DUW0	5.33667	7.00000		
HdrB	D7DTJ3	1.46509	2.41792		
HdrC	D7DTJ4	0.26271	0.83447	−3.1763	0.0005709
HdrB2	D7DTK7	1.26740	0.19729	6.4242	5.04E−05
HdrC2	D7DTK6	0.59715	0.08463	7.0557	9.79E−06
EhaM	D7DQX7	0.24403	0.08458	2.8853	0.0001441
EhaF	D7DQX0	0.13484	0.07181		
EhaH	D7DQX2	0.02553	0.01266	2.0161	0.027129

a A minus sign indicates that the protein was more abundant in Fe(II)/HS^−^.

### Chemical and functional analysis of the proteome.

**FIG 4:**
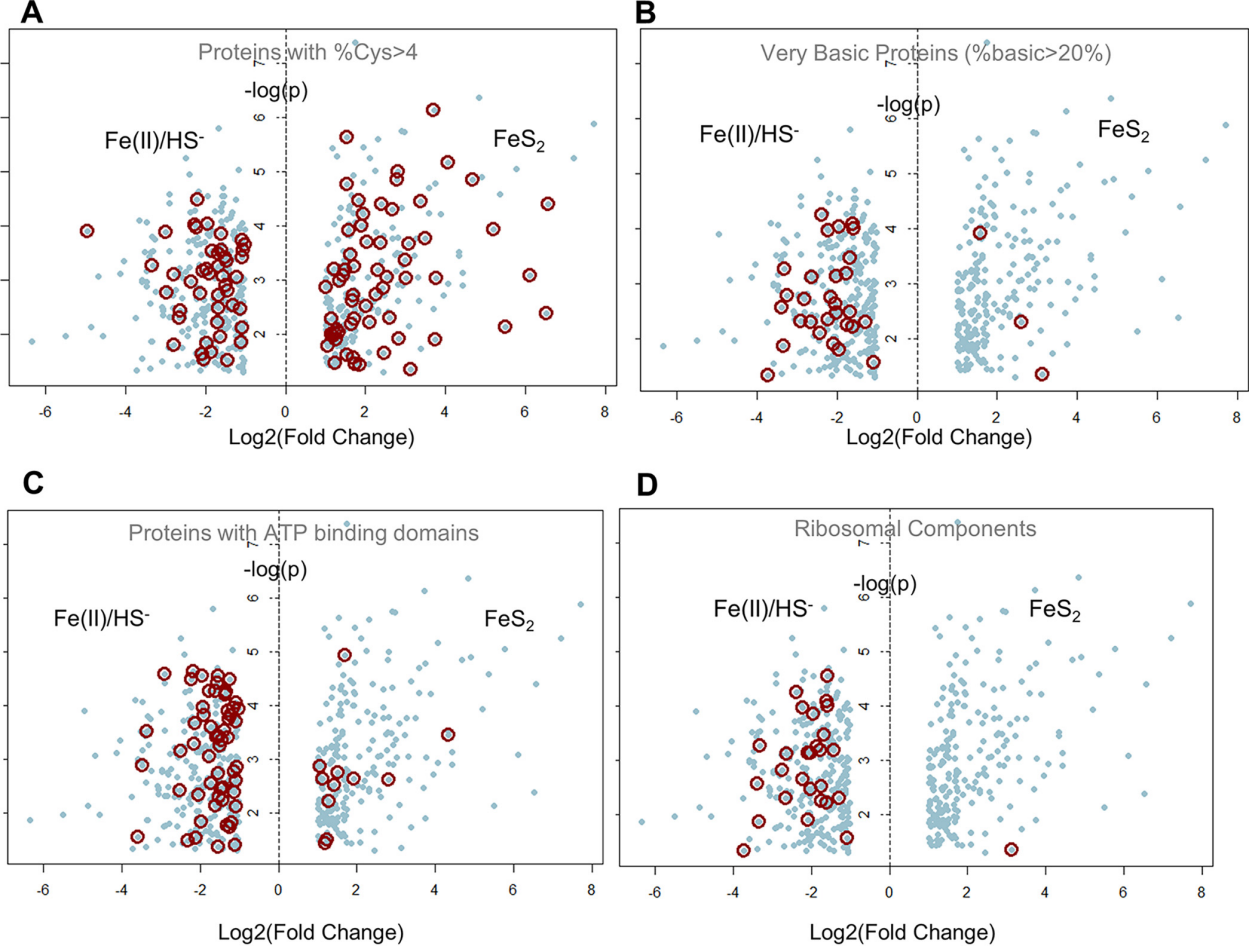
Volcano plot of proteins cultured with FeS^2^ or Fe(II)/HS^−^. Each spot represents a protein, with the fold change (horizontal) and *P* value (vertical) indicated by position in the graph. Circled spots highlight proteins annotated to contain specific sequence and or chemical characteristics. (A) Proteins with high cysteine content. (B) Basic proteins, enriched in lysine, arginine, and histidine. (C) ATP binding domains based on GO categories (GO: 0005524). (D) Structural components of the ribosome. Tabular data for the plots can be found in Table S3a to e.

Biologically mediated reductive dissolution of FeS_2_ results in the production of aqueous Fe-S clusters, which are hypothesized to be directly assimilated to meet the Fe and S demands of methanogen cells (30). Rather than relying strictly on protein annotation, we reasoned that the physical and chemical properties derived from amino acid sequence could be informative, particularly due to the relatively high percentage of unassigned proteins. We first examined the amino acid content of proteins differentially expressed between the two growth conditions to look for enrichment of motifs involved in metal binding and Fe-S cluster coordination. In cells grown on FeS_2_ more cysteine-rich proteins (>4% Cys) were detected in significant amounts compared to cells grown with Fe(II)/HS^−^ (Fig. 4A; Table S3a to e). While we did not detect any significant difference in proteins enriched in acidic residues (Asp and Glu) that could potentially substitute for thiols in binding metal cations such as Fe, we did see a striking number of highly basic proteins (>20% Lys, Arg, or His) that were more abundant in cells grown with Fe(II)/HS^−^ (Fig. 4B). Positively charged polypeptide regions are often found in proteins that bind negatively charged molecules such as nucleotides and nucleic acids. For example, ribosomal proteins, transcription factors, and translational machinery all bind DNA, RNA, and/or nucleotides such as ATP and GTP.

Using GO annotations, we examined proteins involved in ATP binding (GO: 0005524) (Fig. 4C). This category had a large number of proteins that changed abundance, with many more found in the Fe(II)/HS^−^ group. Finally, we singled out structural constituents of the ribosome (GO: 0003735) to gain insight into differences in translation. Proteins with this GO classification were more highly expressed in cells grown on Fe(II)/HS^−^ than in those grown on FeS_2_ (Fig. 4D). This is congruent with the observed profile of highly basic protein enrichment in cells grown on Fe(II)/HS that could theoretically bind nucleic acids or nucleotides. Together, these observations indicate that M. voltae alters the expression of ribosomal and associated proteins when grown on Fe(II)/HS^−^ compared to FeS_2_. This suggests that ribosome composition may be different in the two growth conditions, a finding consistent with recent work on Fe homeostasis (31).

### Iron binding proteins.

M. voltae cells grown with FeS_2_ as their sole source of Fe and S expressed a greater abundance of oxidoreductases (GO: 0016491) and Fe-S binding proteins (GO: 0051536) than cells grown on Fe(II)/HS^−^. This observation prompted further investigation into Fe-S binding proteins expressed in each growth condition. Binding and transport of Fe is facilitated by specific cysteine-rich motifs, which we utilized as a search motif within the differentiated proteins predicted to interact with Fe. A number of proteins with Fe-S cluster-binding motifs were differentially expressed. As an example, we looked at CX_2_CX_2_C ferredoxin motifs (32), for which there were 62 hits in the M. voltae proteome based on genome sequence analysis (Table S4). The proteomics data show that 27 of these were differentially expressed (Table S4). Under FeS_2_ growth conditions, 16 annotated proteins (mostly oxidoreductases) and one of unknown function were more abundant. The majority show high similarity to 4Fe-4S coordinating ferredoxins from methanogens. Mvol_0976 was detected at 69-fold-higher levels under FeS_2_ conditions. This hypothetical ferredoxin-like protein is located downstream from a FeoB protein (Mvol_0975; 14-fold increase in FeS_2_) and upstream from a FeoA protein (Mvol_0977; 211-fold increase in FeS_2_). These observations indicate that Mvol_0976 is a functional protein open reading frame (ORF) that shows higher abundance than the Fe transporter FeoB but much lower expression than FeoA, a regulatory protein that modulates FeoB function, under FeS_2_ growth conditions (33). The location of Mvol_0976 suggests that this protein is part of the Feo operon in M. voltae. A BLAST search (34) demonstrated that this protein is unique to M. voltae. The proteomics data suggest that Mvol_0976 may have a distinct role in iron acquisition when FeS_2_ is the only source of Fe and S. A different set of 11 proteins with ferredoxin motifs were more abundant in the Fe(II)/HS^−^ sample group. Overall, the differential regulation of ferredoxin-motif proteins under FeS_2_ versus Fe(II)/HS^−^growth conditions likely reflects specialized roles in iron-sulfur cluster acquisition and utilization or electron transport in M. voltae.

Of particular interest are several differentially expressed proteins identified as members of the radical *S*-adenosylmethionine (SAM) superfamily (35–37). Of the 36 ORFs in the M. voltae genome that harbor characteristic radical SAM cysteine motifs (36), 19 were differentially regulated, with seven of these being more abundant under FeS_2_ growth conditions and the other 12 being increased under Fe(II)/HS^−^ growth conditions (Table S5). Among those more abundant under FeS_2_ growth conditions was a MiaB-like tRNA modifying enzyme (Mvol_1647). Interestingly, tRNA modification, such as that catalyzed by MiaB, is proposed to be part of a global regulatory mechanism in response to environmental stress conditions (38, 39). This suggests that FeS_2_-based growth could represent a challenge for M. voltae, a finding that is consistent with the recently reported dysregulation of Fe homeostasis in cells grown on FeS_2_ (20). Also increased is the anaerobic ribonucleotide reductase-activating enzyme (Mvol_0042), an essential enzyme for nucleotide metabolism under anaerobic conditions (40). Two other radical SAM proteins that increased with FeS_2_ (Mvol_0698 and Mvol_0696) have unassigned functions, but their genes are located close together on the genome. Both of these radical SAM proteins have cysteine residues in addition to those that coordinate the radical SAM cluster, and these residues could bind additional iron-sulfur clusters.

The genetic context of these two radical SAM proteins suggests they play roles in cofactor biosynthesis. Mvol_0696 exhibits homology to NifB, an enzyme involved in the biosynthesis of the iron molybdenum cofactor in nitrogenase (41, 42). This observation is intriguing, as M. voltae is not a diazotroph and is incapable of fixing nitrogen (i.e., it does not express nitrogenase). Mvol_0698 exhibits homology with the both elongator protein 3, a radical SAM enzyme that contains an accessory domain with histone acetyltransferase activity (43, 44), and MiaB, a methylthiotransferase involved in tRNA modification (45). Moreover, both Mvol_0696 and Mvol_0698 contain domain homology with the SPASM subclass of radical SAM enzymes, which harbor 4Fe-4S auxiliary clusters in C-terminal domain extensions (46). Characterized members of the SPASM subfamily are involved either in modifying ribosomally translated peptides or in the transformation of proteins into active enzymes, such as in the generation of formylglycine (FGly) in arylsulfatase proteins; these enzymes utilize FGly as a cofactor to cleave sulfate monoesters in a variety of substrates (47, 48). Collectively, the genetic context of Mvol_0696 and Mvol_0698 supports the notion that these two radical SAM enzymes play undefined roles in either cofactor biosynthesis or tRNA modification reactions associated with the cellular response due to trafficking FeS_2_ reductive dissolution products.

Five radical SAM enzymes with no functional annotation were found at higher abundance under Fe(II)/HS^−^ growth conditions. Mvol_0045 and Mvol_1681 are both surrounded by ORFs related to nucleotide metabolism. Mvol_1414 is a radical SAM enzyme that uses the less common CX_5_CX_2_C HmdB motif (49) and has homology to the hydrogenase maturation protein HydE (50). Mvol_1348 shows similarity to the [FeFe]-hydrogenase maturation enzymes HydE and HydG, and nearby is an ORF for HypD, the Fe-only hydrogenase (32), suggesting that Mvol_1348 may be involved in cofactor biosynthesis for HypD. This proteomics data reveal that the radical SAM enzymes with significantly different abundance between FeS_2_ and Fe(II)/HS^−^ growth conditions are involved in stress response, nucleotide metabolism, and cofactor biosynthesis.

Two proteins of specific interest that contribute to the differences between the two conditions were a DrsE domain-containing protein (Mvol_0773) and DUF 2193 (Mvol_0354), both higher in the FeS_2_ condition. DrsE domains are involved in intracellular sulfur reduction and interact with desulfoferrodoxin ferrous iron binding proteins (Mvol_0775) (51). The oxidoreductases Mvol_0773 and Mvol_0775 are next to each other in the genome and are both found in significantly greater amounts in the FeS_2_ condition. Interestingly, a cell wall binding protein (Mvol_0771) and an uncharacterized protein (Mvol_0772) were also detected in higher quantities in the FeS_2_ condition. The proximity of these four proteins in the genome suggests that this is an iron- and/or sulfur-regulated operon. In the Fe(II)/HS^−^ condition, an ApbE-like protein (COG2122, Mvol_0331) was more abundant. This protein has been suggested to be essential in sulfide assimilation (52); thus, an increase may be expected to facilitate biosynthesis of cysteine and homocysteine. The DUF2193 protein of unknown function (Mvol_0354) was more abundant in FeS_2_ samples and appears to be well conserved in methanogens. A cluster of conserved CX_2_CX_6_DX_2_(H/C)X_2_C residues near the C termini of the ApbE-like (Mvol_0331) and DUF2193 (Mvol_0354) proteins could act as a ligand for labile Fe-S cluster coordination.

### Conserved proteins and oxidoreductases.

We reasoned that oxidoreductases could be important for mobilizing FeS_2_ {which is composed of ferrous-persulfide units, [Fe(II)S_2_]^0^}, as well as changing the oxidation state and speciation of Fe and S within the cell. In the M. voltae proteome, we identified 33 oxidoreductases (Table S6) that were not clearly associated with a well-defined metabolic pathway—for example, methanogenesis, hydrogenase chemistry, or cellular respiration. Of these, 21 were more abundant on FeS_2_, 18 in the intracellular fraction and 3 extracellularly. Two of these proteins (Mvol_0775 and Mvol_0776) are conserved in two other methanogen species, *M. maripaludis* and Methanosarcina barkeri, recently reported to reduce FeS_2_ (19). Mvol_0775 is annotated as a mononuclear iron-binding desulfoferrodoxin protein, with relatives involved in superoxide reduction to peroxide as part of the cellular antioxidant defense (53), while Mvol_0776 is a carboxymuconolactone decarboxylase similar to peroxiredoxins. These enzymes are mainly distributed in anaerobic archaea and bacteria, including sulfate reducers (54). These enzymes may play a role in antioxidant defense or possibly in reduction of the persulfide unit in FeS_2_. The peroxide and persulfide anions are isoelectronic as well as roughly isostructural; consequently, they may react in a similar fashion. For example in catalases, the peroxide bond undergoes two electron heterologous cleavage, leading to water and a metal-oxo intermediate. An analogous metal-mediated reaction with a persulfide could be drawn, or the attacking electrophile may even be the thiolate side chain of a cysteine. As another possibility, two-electron reduction of the peroxide bond in several symmetrically bridged dimetal-peroxide complexes leads to a pair of high-valence metal-oxo species as well as a similar reaction for a bridging persulfide unit, leading to two metal sulfides (55, 56).

To place the differentiated proteins from M. voltae in evolutionary context, we searched the data for proteins conserved across archaea and bacteria. Utilizing work from our collaborators which chronicled Fe-S proteins and their phylogeny (49), we searched Fe-S proteins and motifs conserved across many species of archaea and bacteria involved in the uptake, trafficking, and storage of Fe and S (57). Eleven of the 41 proteins described by Johnson et al. (57) are annotated in M. voltae (Table S7). Of those 11, 10 were detected in our shotgun proteomic data, four of which were differentiated. Three had higher abundance in the FeS_2_ condition: two FeoA type proteins and FeoB. A HemC-type protein, Mvol_0134, which is a probable porphobilinogen deaminase (PBGD), had higher abundance in the Fe(II)/HS^−^ condition. This probable PBGD protein is likely involved in the production of linear tetrapyrroles, which serve as precursors to a variety of cofactors, including the methanogenesis-associated F_430_ (58). Upregulation of this protein in cells grown on nonmineral sources of Fe and S would be consistent with increased metabolic activity, as described above.

### Membrane proteins.

A critical step in uptake of extracellular material involves membrane transport. While the technical approach used here isolated soluble proteins, 55 membrane or membrane-associated proteins were significantly differentiated between Fe(II)/HS^−^ and FeS_2_ conditions (Table S8). Membrane proteins were categorized by GO annotation from UniProt (29), Pfam (59), and PHYRE (60). PHYRE was used when standard methods failed to assign a functional category. If PHYRE failed to yield results, PSORTb was utilized to predict cellular localization. Of the 55 regulated membrane proteins, 29 were in higher abundance in FeS_2_-grown cells. This included the energy-converting hydrogenase Eha or the (NiFe)-hydrogenase-3-type complex (Mvol_1594), a multisubunit membrane-bound protein which has an essential role in methanogenesis by supplying electrons to anaplerotically reduce CO_2_ to formylmethanofuran (61). FeoB (Mvol_0975; discussed above), a membrane-bound protein involved in ferrous iron uptake and transport, was also significantly more abundant in cells grown on FeS_2_. The protein Mvol_0781, a heavy metal-translocating P-type ATPase, was detected in higher abundance in the FeS_2_ condition and is another candidate for involvement in uptake of ferrous iron or other transition metals. A homolog of this ATPase found in Pseudomonas aeruginosa was shown to be selective for uptake of zinc and copper (62). Two membrane-associated transport proteins were found in higher abundance in cells grown in the Fe(II)/HS^−^ condition, an ABC transporter for molybdenum (Mvol_0749), and EcfA, part of an ABC-transporter complex (Mvol_1619). Other proteins, including the transcriptional regulator TrmB (Mvol_1582) and formate hydrogenlyase subunit 4-like protein (Mvol_1241), were also found in higher abundance in cells grown on Fe(II)/HS^−^. TrmB is a control point for sugar metabolism (63), and formate hydrogenlyase is a central enzyme in anaerobic metabolism (64), contributing to the reasoning that M. voltae has the potential to generate more ATP and reducing equivalents, an indication of an active metabolism under Fe(II)/HS^−^ culture conditions.

### Extracellular proteins.

**FIG 5:**
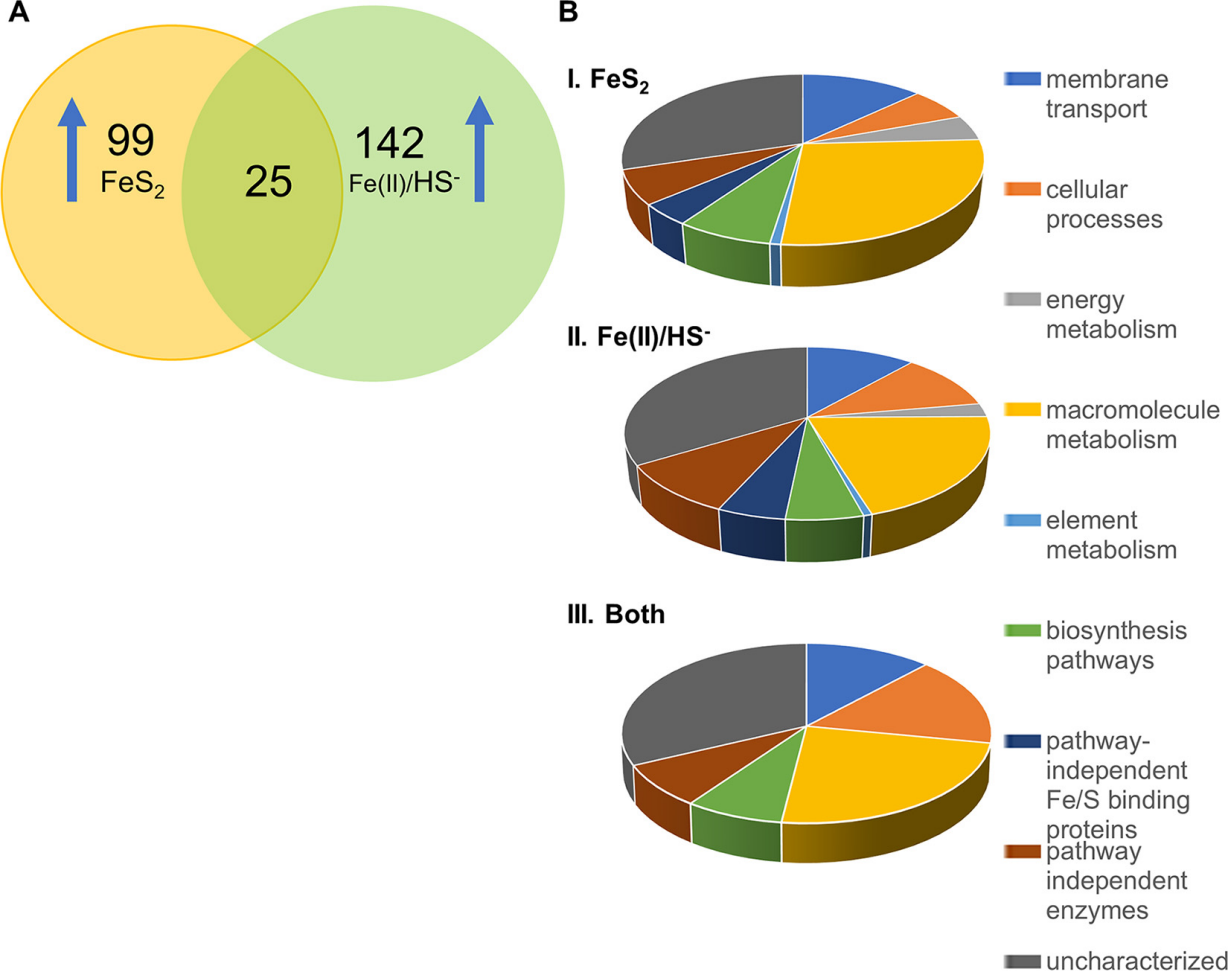
Extracellular protein pools. (A) Comparison of proteins present in the media under different growth conditions. Proteins unique to a condition were at least 20-fold more abundant in that condition. (B) Functional categorization of extracellular proteins upregulated during growth with FeS^2^, Fe(II)/HS^−^, and both. Upregulated proteins were functionally categorized according to their UniProt annotations and GO classifications. While the functional distributions of proteins from the sulfide and pyrite conditions were similar, proteins specifically upregulated specifically by one or the other growth condition were predominantly in the “pathway independent Fe/S binding proteins,” “energy metabolism,” and “element metabolism” groups (“pathway independent” indicates that the proteins could not be clearly identified with a particular metabolic pathway or process). Further division of each group into subgroups is given in Table S12.

In order to address the possibility that M. voltae may excrete essential enzymes targeted at the FeS_2_ reductive dissolution process, we analyzed the media for proteins that could facilitate mineral reduction and metal transport. After careful removal of cells to avoid lysis, acetone precipitation was used to collect proteins from the extracellular fraction. A tiered approach was used to compare the FeS_2_ and Fe(II)/HS^−^ extracellular proteomes. First, the abundances of individual proteins were compared between intra- and extracellular fractions in each condition. We focused only on proteins that were highly enriched in the extracellular fraction (FC > 20; *P* < 0.05). This step was taken to eliminate proteins found extracellularly due to minor cell lysis rather than active excretion. The filtered lists of proteins enriched in the extracellular fractions from each condition were then compared. This yielded 25 proteins that were enriched in the extracellular fractions of both conditions, 99 proteins specific to FeS_2_-grown cells, and 142 distinct to the Fe(II)/HS^−^ condition (Fig. 5; Tables S9 to S11). Of the 25 proteins present in both conditions, 6 are uncharacterized, two are membrane associated (Mvol_0383 and Mvol_0341), and two are transferases (Mvol_1039 and Mvol_0237) (Table S11).

Extracellular proteins enriched in only one condition were grouped by functional annotation for comparison. Of the proteins differentially expressed between the two growth conditions, 84 were annotated as uncharacterized and were investigated using PHYRE (Table S13). Proteins that were more abundant in the extracellular milieu of cells grown on Fe(II)/HS^−^ included four radical SAM proteins, 45 uncharacterized proteins, and 9 transport proteins (Fig. 5; Table S12). Overall, proteins enriched in the FeS_2_ extracellular conditions were involved in membrane transport, sulfur metabolism, oxidoreductases, and uncharacterized (putative) Fe-S binding (Fig. 5B). More specifically, three radical SAM 4Fe-4S proteins (Mvol_0826, Mvol_1151, and Mvol_0698) and one 4Fe-4S ferredoxin iron-sulfur binding domain protein (Mvol_0878) were more abundant in the extracellular fraction of FeS_2_-grown cells. A cysteine-rich protein, Mvol_1221, and the periplasmic copper-binding protein (Mvol_0646) were also present in high abundance in the FeS_2_ extracellular fraction.

## DISCUSSION

**FIG 6:**
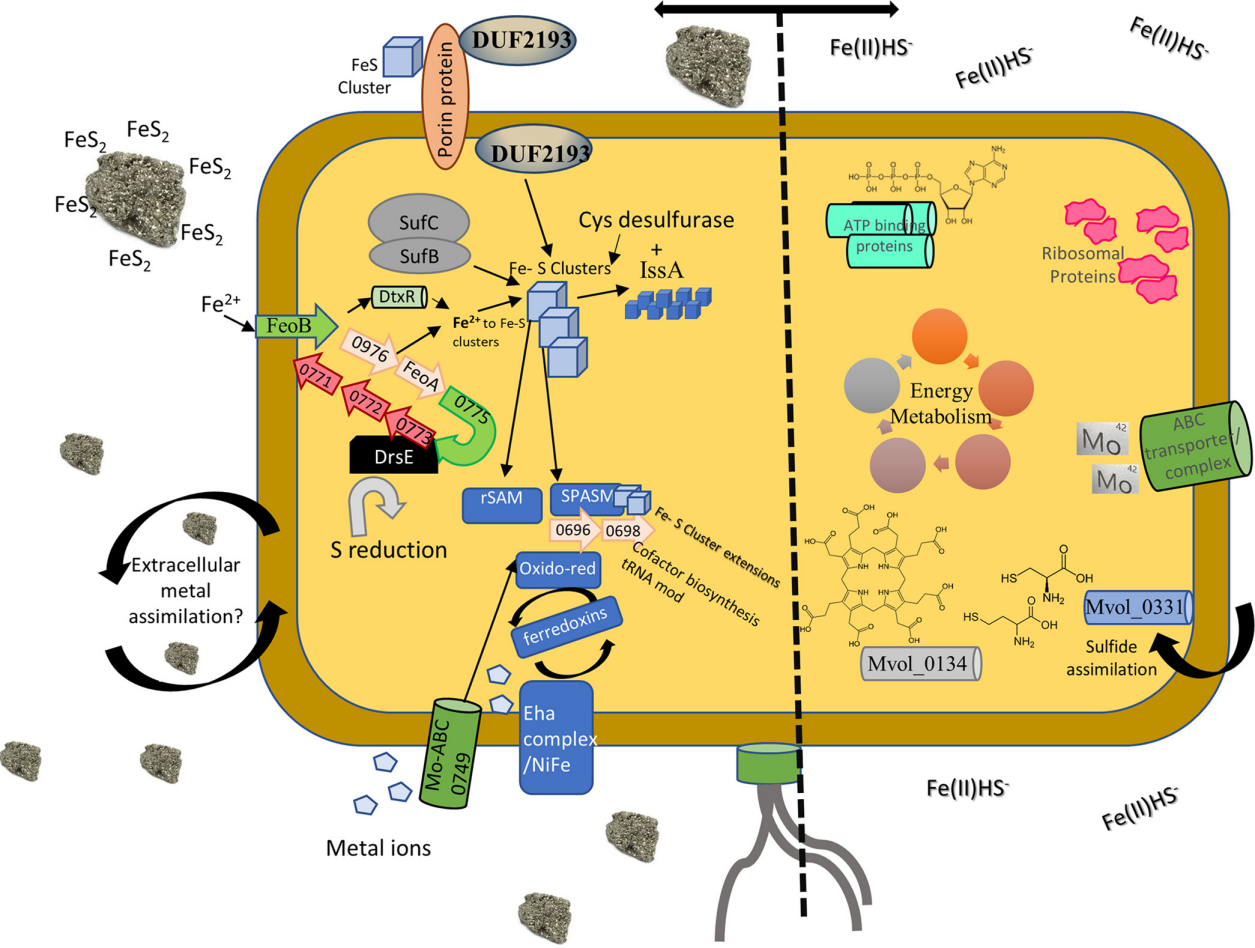
Overview of proteins and pathways associated with M. voltae grown on pyrite (left) or Fe(II)HS^−^(right). Potential mechanisms for Fe-S cluster import and proteins involved, such as DUF2193, the iron uptake protein FeoB, and potential Fe-S extracellular metal binding and assimilation targets. Iron and sulfur usage through desulfurases, ferredoxins, and the SufC and SufB proteins is also represented. On the right, increased energy metabolism, ATP-binding proteins, and the ABC molybdenum transporter Mvol_0749 are represented. Sulfide assimilation into cysteine, homocysteine, and tetrapyrrole formation is also depicted.

This comparative shotgun proteomics analysis of M. voltae grown in the presence of mineral or nonmineral sources of Fe and S generated a deep view into the expression patterns of proteins and pathways. The coverage and depth of this analysis allowed us to construct a view of what happens intracellularly, under each condition, to assimilate iron and sulfur (Fig. 6). With 1,658 predicted protein-coding genes, this methanogen has fewer than half of the genes of a typical strain of E. coli. As one might expect for an organism with a petite genome, a high percentage of the proteome would be expected to be translated under any given circumstance. In this case, 1,269 (77%) of the predicted ORFs were detected. The first noteworthy clue that the form of Fe and S is critical to this organism is that 509 (40%) of the measured proteins had a significantly different abundance (FC > 2; *P* value < 0.05) between sample groups. This is a dramatic response for an archaeal species compared with other environmental pressures such as viral infection and acute oxidative stress (65, 66). While the shift in the expressed proteome was robust when the two growth conditions were compared, the response was balanced; with 285 proteins more abundant in Fe(II)/HS^−^-grown cells and 224 proteins more abundant in cells provided mineral FeS_2_ (Fig. 2). The large yet balanced response suggests that M. voltae cells undergo substantial physiological adjustments that involve a range of functions based on the available Fe and S source (Fig. 6).

We began our analysis by looking at central metabolic pathways. Proteins associated with amino acid, protein and nucleic acid metabolism were generally more highly expressed in cells grown on Fe(II)/HS^−^ than in FeS_2_-grown cells. Similarly, proteins related to respiration, membrane transport, cofactor biosynthesis, nucleic acid metabolism, and biosynthesis of TCA intermediates were also more abundant in Fe(II)/HS^−^-grown cells. Interestingly, a large number of ribosomal proteins showed differential abundance between the growth conditions (Fig. 1, 3, and 6). Our data are consistent with recent work demonstrating that bacteria alter the expression of ribosomal proteins and the composition of ribosomes based on Fe availability (31). The modest change in methanogenesis-related proteins, along with previous work which reported that methanogenesis rates are nearly unchanged (19), suggests no direct connection between this cellular process and either growth condition. In contrast, proteins that potentially have roles in Fe and S transformation, including metal uptake, iron-sulfur trafficking and storage proteins, transcriptional regulators, and oxidoreductases, were detected in significantly higher abundance in the FeS_2_-grown cells.

We sought to test the hypothesis that a specialized set of proteins would be required for growth of M. voltae provided with FeS_2_ as the sole form of Fe and S. Metal binding activity would be requisite in such a protein pool; therefore, we began by querying the data for proteins with the cysteine-rich ferredoxin motif CX_2_CX_2_C. We detected 27 of the 62 CX_2_CX_2_C domain-containing proteins in the genome at differential abundances dependent on the growth condition. Of the regulated proteins, 16 were increased in the presence of FeS_2_. Proteins with predicted involvement in metal binding and transport were of primary interest, like the FeoAB pair (Mvol_0977 and Mvol_0975) and the associated transcription factor DtxR (Mvol_0620). DtxR is a transcriptional regulator involved in maintaining transition metal homeostasis (67–69). It has been shown in Pyrococcus furiosus that when Fe(II) is low in abundance and unavailable to DtxR, this protein binds the promoter of FeoAB and induces expression (68). When DtxR binds Fe(II), it suppresses its own expression and that of FeoAB. The increased expression of DtxR and FeoAB could imply that the cells sense Fe(II) limitation when grown with FeS_2_ (as discussed in reference 20). Another protein of interest was an uncharacterized protein that contains DUF2193 (Mvol_0354). This protein of unknown function has metal binding motifs, was more abundant in FeS_2_ samples, and was found to be highly conserved in methanogens. Oxidoreductases could also play a direct role in growth by either reducing FeS_2_ or being involved in FeS_aq_ trafficking from the reduced mineral surface to the cell. Thirty-three were identified, with 21 of these being more abundant in the FeS_2_ samples (Table S6). Due to the differential abundance in the FeS_2_ condition and the presumed 4Fe-4S binding sites, these oxidoreductases, three of which were enriched in the extracellular fractions, are interesting targets for further investigation.

Nineteen radical SAM enzymes were significantly different between conditions. Among those significantly increased in FeS_2_ growth conditions was a MiaB-like tRNA modifying enzyme. Interestingly, the tRNA modification catalyzed by MiaB is proposed to be part of a global regulatory mechanism in response to environmental stress conditions (38, 39). This is consistent with the suppression of proteins in growth-associated pathways in the FeS_2_ growth condition compared to the Fe(II)/HS^−^ condition. As a final point, the role of differentially regulated radical SAM proteins in the extracellular fractions remains to be elucidated.

M. voltae cells require direct access to FeS_2_ in order to catalyze its reduction, or an extracellular protein complex of >100 kDa is involved in this process (20). To screen for extracellular proteins potentially involved in FeS_2_ reduction, we investigated differences in the extracellular proteome using strict criteria in which protein abundance had to be enriched extracellularly compared to the intracellular fraction, to rule out cell lysis during culture and/or handling as an explanation. The extracellular fractions were surprisingly distinct when conditions were compared, with far more proteins unique to a condition than shared (Fig. 4; Tables S9 to S11). The identified groups are populated with radical SAM, oxidoreductases, and putative Fe-S binding proteins, providing a short list for further investigation. Archaea are known to secrete large numbers of extracellular vesicles (70). It is intriguing to postulate that this process could be used to deliver proteins extracellularly for uptake and transport of Fe and S. Analysis of the 25 proteins enriched in extracellular fractions under both growth conditions returned two proteins with canonical archaeal export signals (Mvol_0838 and Mvol_0569), indicating that additional mechanisms are at work (71). At this time, it remains unclear if M. voltae actively secretes proteins of >100 kDa to facilitate reductive dissolution of FeS_2_ and/or acquisition of soluble Fe and/or S species.

Proteins such as oxidoreductases, Fe-S binding proteins, and radical SAM enzymes could all have roles in the multistep process that begins with FeS_2_ reduction and the trafficking of the reduction products into the cell (Fig. 6). These could then be stored in the cell by other classes of proteins using either cysteine-rich motifs or electrostatic interactions, as has been observed for the IssA protein in Pyrococcus furiosus (27). Even though we did not specifically target integral membrane proteins, the increased abundance of FeoAB and other annotated transporters in the FeS_2_ condition supports the hypothesis that M. voltae has specialized protein machinery facilitating the reductive dissolution and subsequent assimilation of Fe and S directly from FeS_2_.

This study establishes a foundation on which to base characterization of the pathways and proteins responsible for Fe and S acquisition, trafficking, and storage in the model methanogen M. voltae. The ability of M. voltae to utilize mineral and nonmineral sources of Fe and S provides unique opportunities to study the mechanisms employed for acquiring, trafficking, and storing these elements. Knowledge along these lines will undoubtedly open new avenues of Fe and S biochemistry and yield valuable industrial and biotechnological insights with applications related to metal sequestration, processing, and biomining.

## MATERIALS AND METHODS

### Cell culture conditions.

M. voltae strain A3, obtained from the American Type Culture Collection (ATCC BAA-1334), was grown in Fe- and S-free basal medium that contained the following (in grams per liter): NaCl, 21.98; MgCl_2_·6H_2_O, 5.10; NaHCO_3_, 5.00; NH_4_Cl, 0.50; K_2_HPO_4,_ 0.14; KCl, 0.33; CaCl_2_·2H_2_O, 0.10. The basal medium was amended with 0.01 g L^−1^ Fe(NH_4_)_2_(SO_4_)_2_·6H_2_O and 0.480 g L^−1^ Na_2_S·9H_2_O for Fe (II)/HS^−^-grown cells. Thirty minutes prior to inoculation, sulfide was added from an anoxic, sterile stock. Basal medium was amended with a synthetic FeS_2_ slurry to 2 mM Fe for FeS_2_ cultures (19). Trace element, vitamin, and organic solutions were added to the basal medium (each 1% [vol/vol]), based on the work of Whitman et al. (72), but Fe was omitted, and sulfate salts were replaced with chloride salts at the same molar concentrations. The trace element solution contained the following (in grams per liter): nitriloacetic acid, 1.500; MnCl_2_·4H_2_O, 0.085; CoCl_2_·H_2_O, 0.100; ZnCl_2_, 0.047; CuCl_2_·2H_2_O, 0.0683; NiCl_2_·6H_2_O, 0.0683; Na_2_SeO_3_, 0.200; Na_2_MoO_4_·2H_2_O, 0.100; and Na_2_WO_4_·2H_2_O, 0.100. The vitamin solution contained the following (in grams per liter): pyridoxine HCl, 0.01; thiamine HCl, 0.005; riboflavin, 0.005 g; nicotinic acid, 0.005; calcium D(+) pantothenate, 0.005; biotin, 0.002; folic acid, 0.002; and cobalamin, 0.0001. The organics solution consisted of 1 M sodium acetate·3H_2_O, 75 mM l-leucine HCl, and 75 mM l-isoleucine HCl. M. voltae cultures were supplemented with a 40% (wt/vol) sodium formate stock solution added to a final concentration of 0.4% (vol/vol) prior to inoculation. The pH of the medium was set to 7.0 before autoclaving in an 80:20 N_2_-CO_2_ headspace. After autoclaving and the addition of amendments, the pH was 7.2. The pH of the medium increased during growth, reaching maxima of 7.7 and 7.8 in stationary-phase cultures for the pyrite and sulfide conditions, respectively. Sulfide was provided in excess of cellular demands (2 mM) and did not change appreciably during growth of the cells. Acid-volatile Fe(II) was found to decrease from the added 26 μM of Fe(NH_4_)_2_(SO_4_)_2_ to 11 μM Fe(II) in cultures that had reached stationary phase, indicating that Fe and S are not limiting in the Fe(II)/HS^−^-grown cells.

### Cultivation procedures.

Seventy-five-milliliter cultures of M. voltae were grown in 165-mL serum bottles and were harvested during mid-log-phase growth. Anaerobic conditions were maintained during culture and harvesting of cells. Samples were centrifuged at 4,696 × *g* for 20 min at 4°C in a swinging-bucket rotor. For extracellular fractions, 10 mL of culture supernatant was decanted under aerobic conditions into 40 mL of ice-cold 100% acetone (Fisher Scientific, Fair Lawn, NJ) and left at −20°C for 4 h. The samples were then centrifuged to pellet the extracellular proteins and stored at −80°C.

### Protein extraction.

Cell pellets were resuspended in 500 μL of pH 7 phosphate buffer (137 mM NaCl, 2.7 mM KCl, 10 mM Na_2_HPO_4_, 1.8 mM KH_2_PO_4_) with protease inhibitor mix (complete mini EDTA-free protease inhibitor cocktail; Roche). Samples were lysed using an ultrasonic homogenizer on ice for 15 min and were then centrifuged, leaving the soluble protein fraction in the supernatant. The supernatant was collected, four column volumes of ice-cold 100% acetone was added to precipitate the proteins, and samples were placed at −80°C for 1 h and then at −20°C overnight. The acetone was decanted, and protein pellets were stored at −80°C for proteomics analysis.

### Proteomics analysis.

Protein pellets were digested using a Thermo Scientific EasyPep mini MS sample prep kit (catalog no. A40006). Briefly, samples were reduced and alkylated using iodoacetamide and digested with a mixture of trypsin/LysC, a modified version of that described by Lundby et al. (73). Samples were passed over a C_18_ reverse-phase column prior to LCMS to remove undigested protein. LCMS was performed on an UltiMate 3000 RSLCnano system (Thermo Scientific, San Jose, CA) using a self-packed ReproSil-Pur C_18_ column (100 μm by 35 cm) packed at 9,000 lb/in^2^ using a nano-LC column packing kit (nanoLCMS Solutions, Gold River, CA). The two-component solvent system used a gradient from 2 to 90% B over 92 min. Solvent A was water with 0.1% formic acid, and solvent B was acetonitrile with 0.1% formic acid. The LC was coupled to the mass spectrometer by a digital Pico View nanospray source (New Objectives, Woburn, MA) that was modified with a custom-built column heater and an active background ion reduction device (ABIRD) background suppressor (ESI Source Solutions, Woburn, MA). Data-independent acquisition (DIA) mass spectral analysis was performed using an Orbitrap Fusion mass spectrometer (Thermo Scientific, San Jose, CA). Six gas-phase fractions (GPF) of the biological sample pool were used to generate a reference library. The GPF acquisition used 4 *m/z* precursor isolation windows in a staggered pattern (GPF1, 398.4 to 502.5 *m/z*; GPF2, 498.5 to 602.5 *m/z*; GPF3, 598.5 to 702.6 *m/z*; GPF4, 698.6 to 802.6 *m/z*; GPF5, 798.6 to 902.7 *m/z*; GPF6 898.7 to 1,002.7 *m/z*). Biological samples were run on an identical gradient as the GPFs using a staggered window scheme (4 *m/z* with an Exploris 480 instrument; 24 *m/z* with a Fusion instrument) over a mass range of 385 to 1,015 *m/z*. An empirically corrected library which combines the GPF and the deep neural network Prosit (74) were used to generate predicted fragments and retention times using Scaffold DIA (Proteome Software, Portland, OR).

### Data analysis.

DIA data were analyzed using Scaffold DIA (2.1.0). Raw data files were converted to mzML format using ProteoWizard (3.0.19254) (75). Deconvolution of staggered windows was performed. Analytical samples were aligned based on retention times and individually searched against uniprot-M_Voltae_UP000007722_20200218.fasta.z3_nce33_v2.dlib with a peptide mass tolerance of 10.0 ppm and a fragment mass tolerance of 10.0 ppm. Variable modifications considered were limited to cysteine. Tryptic peptides with a maximum of 1 missed cleavage site were allowed. Only multiply charged peptides from 6 to 30 amino acids were considered (76). Peptides identified in each sample were filtered by Percolator (3.01.nightly-13-655e4c7-dirty) (77–79) to achieve a maximum false discovery rate (FDR) of 0.01. Individual search results were combined, and peptide identifications were assigned posterior error probabilities and refiltered to an FDR threshold of 0.01 by Percolator. Peptide quantification was performed using Encyclopedia (0.9.2). For each peptide, the 5 highest-quality fragment ions were selected for quantitation. Proteins that contained similar peptides and could not be differentiated based on MS/MS analysis were grouped to satisfy the principles of parsimony. Proteins with a minimum of 2 identified peptides were thresholded to achieve a protein FDR of 1.0%.

### Statistical analysis.

Data from Scaffold DIA (Proteome Software, Inc.) represent average protein intensity, which takes into account all peptide intensities assigned to a protein. The intensities for data analyzed with the Scaffold DIA software were log_10_ transformed and normalized using the standard Scaffold settings. Subsequent analysis was performed using Excel and bioinformatics tools: Metaboanalyst (80), PHYRE (60), and PSORTb (81). For Metaboanalyst, data spreadsheets were first uploaded and checked for integrity. Protein abundance values were interquartile range (IQR) filtered to eliminate outliers. Missing features were replaced using the KNN algorithm, and features with more than 50% missing values were discarded. Protein abundances were then normalized by the sum of all features within a sample, log transformed, and autoscaled (μ centered, divided by the standard deviation of each variable) prior to statistical analysis. *t* test and fold change analyses were performed to assess significance and magnitude of protein abundance differences (volcano plot). The heat map employed Ward’s method of hierarchical clustering based on Euclidean distances calculated for samples and features. Feature were ranked by *t*-test values.

### Data availability.

Raw and processed proteome data can be found at the ProteomeXchange Consortium via the PRIDE partner repository under the data set identifier PXD024933. This data set has been referenced by Payne et al. (20).
